# Recent developments in *CrystFEL* 1

**DOI:** 10.1107/S1600576716004751

**Published:** 2016-03-29

**Authors:** Thomas A. White, Valerio Mariani, Wolfgang Brehm, Oleksandr Yefanov, Anton Barty, Kenneth R. Beyerlein, Fedor Chervinskii, Lorenzo Galli, Cornelius Gati, Takanori Nakane, Alexandra Tolstikova, Keitaro Yamashita, Chun Hong Yoon, Kay Diederichs, Henry N. Chapman

**Affiliations:** a Centre for Free-Electron Laser Science, Deutsches Elektronen-Synchrotron DESY, Notkestrasse 85, 22607 Hamburg, Germany; b Department of Biology, Universität Konstanz, Box 647, 78457 Konstanz, Germany; c Moscow Institute of Physics and Technology, 141700 Moscow, Russian Federation; d Department of Biological Sciences, Graduate School of Science, The University of Tokyo, 7-3-1 Hongo, Bunkyo-ku, Tokyo 113-0033, Japan; e Department of Physics, University of Hamburg, Luruper Chaussee 149, 22761 Hamburg, Germany; f RIKEN SPring-8 Center, Sayo, 679-5148, Japan; g Centre for Ultrafast Imaging, Luruper Chaussee 149, 22761 Hamburg, Germany

**Keywords:** data processing, serial crystallography, X-ray free-electron lasers, XFELs, computer programs

## Abstract

*CrystFEL* is a suite of programs for processing data from ‘serial crystallography’ experiments, which are usually performed using X-ray free-electron lasers (FELs) but also increasingly with other X-ray sources. The *CrystFEL* software suite has been under development since 2009, just before the first hard FEL experiments were performed, and has been significantly updated and improved since then. This article describes the most important improvements which have been made to *CrystFEL* since the first release version. These changes include the addition of new programs to the suite, the ability to resolve ‘indexing ambiguities’ and several ways to improve the quality of the integrated data by more accurately modelling the underlying diffraction physics.

## Abstract 2

Developments in the *CrystFEL* software suite, for processing diffraction data from ‘serial crystallography’ experiments, are described.

## 1. Introduction   

*CrystFEL* is a software suite created to address the processing needs of serial femtosecond crystallography (SFX). It has been under development since 2009 and was first made publicly available in 2012 (White *et al.*, 2012 ▸). It has become the most widely used software for this purpose, with over 40 journal articles describing significant use. Many of these were performed with no involvement whatsoever from the developers or ‘core’ advanced users. Alongside free-electron laser (FEL) experiments, *CrystFEL* is equally applicable to serial crystallography (SX) experiments performed using synchrotron light sources (Stellato *et al.*, 2014 ▸; Nogly *et al.*, 2015 ▸).

The first release version of *CrystFEL* was 0.3.0, which was preceded by several internal test versions. The current version is 0.6.1, which was released in August 2015. Between these versions, many changes and improvements have been made to *CrystFEL*, not only as a result of developments in SFX data processing techniques, but also to improve the user interface, stability and consistency. *CrystFEL* is a free and open-source software project, meaning that contributions in the form of changes to the source code can easily be made from outside the core group of developers. This has already taken place several times since *CrystFEL* was first published, and such contributions are actively encouraged.

The purpose of this article is to describe some of the most important changes between versions 0.3.0 and 0.6.1 of *CrystFEL*. §2 of this article describes the changes made to the range of programs included in the suite, and §3 concerns a technical improvement to *CrystFEL*’s way of handling input data. The remaining sections are directly related to the issues of data quality in serial crystallography, an area that has seen very rapid development in the past few years. One aspect of this is the ability to resolve ‘indexing ambiguities’, which have been a problem since the very first SFX experiments (Chapman *et al.*, 2011 ▸). The algorithm available in *CrystFEL* for this purpose is slightly different from those previously described in the literature, and therefore it is described in detail in §4. §§5 and 6 describe progress in modelling the underlying diffraction process more accurately, which results in an improvement to the final merged data quality.

## 2. Changes to the available programs   

Six new programs have been added to the *CrystFEL* suite, and two have been removed. The following programs are new:

*ambigator*, a program for resolving indexing ambiguities, described in §4.

**Figure 1:**
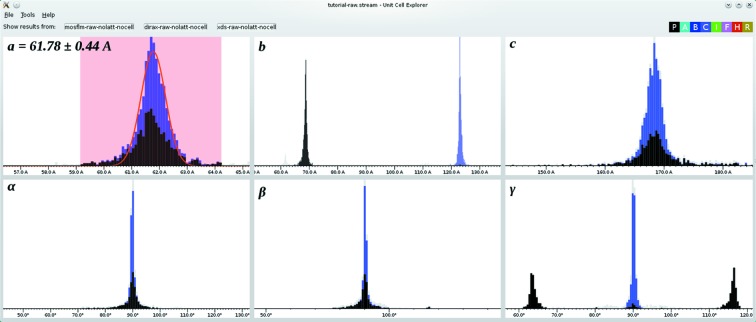
Screenshot of *cell_explorer* in use, showing a first indexing run on a sample (runs 130–139) of the previously published data for the 5-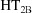 receptor (Liu *et al.*, 2013 ▸). Two alternative sets of lattice parameters, in this case with different centring types, have been found, which in this example represent the same lattice. A Gaussian function has been fitted to the distribution of *a* parameters, yielding a mean and standard deviation.

*cell_explorer*, a graphical tool for unit-cell determination. A run of *indexamajig* without the unit-cell matching procedure (White *et al.*, 2012 ▸) produces a list of unit-cell parameters independently determined from each one of the diffraction patterns. The program *cell_explorer* displays histograms for *a*, *b*, *c*, α, β and γ which the user can zoom and drag as well as allowing the size of the histogram bins to be altered. Different centring types are represented by colour. The user can select a range for any of the parameters, in which case the other histograms will change to highlight only the crystals having the parameter within the specified range. A Gaussian function can be fitted to the distribution of parameters within the selected range (as shown in Fig. 1 ▸). In this way, quantitative values including error estimates can be calculated for the cell parameters even in the presence of large amounts of contamination from incorrectly indexed patterns, contamination with other crystal forms and alternative indexing options for the same lattice.

*geoptimiser*, a tool for automatically refining multi-panel detector geometry by comparing observed peak locations with the positions calculated for reflections after indexing. The algorithm is broadly similar to that described by Hattne *et al.* (2014 ▸), but additionally allows the possibility of refining in-plane rotations and out-of-plane shifts. This tool is described in detail elsewhere (Yefanov *et al.*, 2015 ▸).

*list_events*, a tool for manipulating lists of diffraction patterns as input for *indexamajig*, described in §3.

*partial_sim*, a simulation tool which complements *pattern_sim* by calculating partial reflection intensities using a geometrical model of Bragg diffraction. This program was described earlier (White *et al.*, 2013 ▸; White, 2014 ▸).

*whirligig*, which analyses *CrystFEL* output ‘streams’ and searches for runs of crystals in similar orientations. This tool is intended for use in experiments similar to that described by Gati *et al.* (2014 ▸), in which diffraction patterns were acquired while performing helical scans of a grid containing microcrystals. A helical scan, as its name suggests, couples rotation of the grid with its linear translation in a particular direction. Wedges of data were identified as coming from single crystals if the orientations of adjacent crystals, as determined by *CrystFEL*, were similar. These wedges were then used for further processing using conventional rotation-data integration software, thereby taking advantage of both the ‘serial’ and ‘rotation’ aspects of data collection.

The following programs also existed in previous versions:

*check_hkl*, which calculates figures of merit based on a single set of reflection data, such as mean 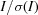 values and completeness.

*compare_hkl*, which calculates figures of merit based on two sets of reflection data, such as *R* factors and correlation coefficients.

*get_hkl*, which is a tool for performing a variety of less common manipulations on reflection data such as expanding symmetry equivalents or adding noise for test purposes.

*hdfsee*, an image viewer that works with *CrystFEL*’s understanding of image data and detector geometry (see §3).

*indexamajig*, the main indexing and integration tool.

*partialator*, a program for merging reflection data with scaling and post-refinement. This program is described further in §6.

*pattern_sim*, a program for simulating image data.

*process_hkl*, a program for merging reflection data using less sophisticated methods than *partialator* (also described in §6).

*render_hkl*, which renders the intensities of reflections in sections through reciprocal space.

The programs *sum_stack* and *powder_plot*, which were available in previous versions, were removed from *CrystFEL*. These programs, respectively, added detector frames to form a ‘virtual powder pattern’ and calculated one-dimensional line profiles from them. We found that their functionality fits more logically in the ‘hit-finding’ software, as described in the next section.

## 3. ‘Multi-event’ input files   

Recent advances in crystallography, including but not limited to the development of serial crystallography, have brought with them the challenges of managing large datasets. In many types of serial crystallography experiment including the ‘injector-based’ approach (Chapman *et al.*, 2011 ▸), the detector is read out repeatedly as crystals are moved into the path of the X-ray beam. As a result, not every image read out from the detector contains a crystal diffraction pattern. To reduce the data volume, and also to convert the image data from any facility-specific data format to a common format, a ‘hit-finding’ stage is first performed in which the crystal diffraction patterns are identified and written to new files. Software able to perform this task includes *Cheetah* (Barty *et al.*, 2014 ▸) and *CASS* (Foucar *et al.*, 2012 ▸). *CrystFEL* has been designed to read data after this type of processing, which has the added benefit of freeing *CrystFEL* from dependence on facility-specific frameworks necessary to access the original data streams (Damiani *et al.*, 2016 ▸).

Much of the FEL data analysis ‘ecosystem’ (which includes applications aside from crystallography, such as spectroscopy and coherent diffractive imaging) has been standardized on the Hierarchical Data Format version 5 (HDF5; http://www.hdfgroup.org/HDF5/) as a facility-independent data interchange format. *CrystFEL*, like both *Cheetah* and *CASS*, follows this convention. This format allows chunks of data, which can be scalar values or arrays of any dimensionality and size, to be organized in a tree-like structure. A single file can therefore contain the image data alongside metadata such as the X-ray wavelength and detector distance. *CrystFEL* has been designed from the start to avoid imposing restrictions on the exact layout of the HDF5 files. Instead, it allows the upstream processing steps performed by the facility analysis framework or hit-finding software to set the file layout, and reads a description of the data layout, as well as how the data in the file relate to pixels in physical space, from a ‘geometry file’. In this way, *CrystFEL* is not bound to any particular ‘flavour’ of HDF5.

**Figure 2:**
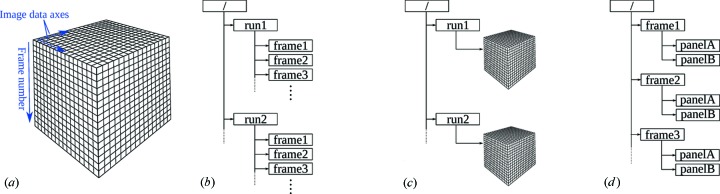
Possible layouts for image data in a ‘multi-event’ HDF5 file. (*a*) A three-dimensional array where one direction corresponds to image data. The correspondence between the dimensions of the array and the image data/frame number axes is arbitrary and can be defined by the user. (*b*) A tree of HDF5 groups, where each frame contains a simple two-dimensional array. (*c*) A combination of the two, where the file contains multiple three-dimensional blocks, each containing many frames of data. (*d*) A tree of HDF5 groups where the data for each of the two detector panels are stored in separate two-dimensional arrays.

Since version 0.6.0, *CrystFEL* can accommodate HDF5 files that contain more than one detector readout event per file. Previously, each individual input file had to contain only one detector frame, resulting in a need for a very large number of small files and therefore inefficient handling by computer filing systems. With a ‘multi-event’ HDF5 file, the detector frames can be grouped together into one large file, which is more efficient and easier to manipulate, transfer across networks and back up. Several possibilities for laying out a multi-event HDF5 file can be accommodated: a three-dimensional array where one dimension represents frame number and the other two the detector readout coordinates, a network of HDF5 ‘groups’ (analogous to familiar filesystem directories), or a combination of the two. These two basic possibilities are illustrated alongside a more complicated one in Figs. 2 ▸(*a*)–2 ▸(*c*). In addition, there is no longer a requirement that the data for all panels of a segmented detector are stored in the same data block (Fig. 2 ▸ *d*), allowing for more flexibility in the layout of the HDF5 file.

Our aim was to accommodate any ‘reasonable’ layout, without adding too much complexity. Many formats are well accommodated by this change, including the native HDF5 layouts used by the Linac Coherent Light Source (LCLS) and SPring-8 Angstrom Compact Laser (SACLA) data acquisition systems, files output by the newest generation of fast counting detectors such as EIGER (Bernstein *et al.*, 2014 ▸), and the native format of the Coherent X-ray Imaging Data Bank (CXIDB; Maia, 2012 ▸). The hit-finding program *Cheetah* (Barty *et al.*, 2014 ▸) can output files directly in a multi-event format based on the format used by the CXIDB. To help enable *CrystFEL* to be easily used on data from an even wider range of sites, we have made some tools available *via* the *CrystFEL* web site for converting CBF, MarCCD and raw image data to HDF5 format.

Whereas previously the input for *indexamajig* consisted of a list of individual filenames to process, the input can now consist of a much smaller list of files, since each file can now contain very large numbers of frames. The new program *list_events* can expand the short list of multi-event files into a much longer list of ‘event descriptors’. This list can then be filtered, sorted or otherwise modified to provide the input for *indexamajig*, which can then process only the events of interest instead of the entire dataset. The list of events could also be used as a basis for a splitting list for *partialator* (§6).

## 4. Resolution of indexing ambiguities   

Indexing ambiguities were a significant problem in serial femtosecond crystallography for the first few years. Certain crystal symmetries permit multiple ways of indexing the lattice which are not equivalent with respect to the true symmetry of the intensities. The situations that permit this include the cases where twinning is possible by merohedry with a rotational twin law, as well as several other cases that occur if the metric symmetry happens to be higher than the true symmetry (White *et al.*, 2013 ▸). In principle, it should be possible to make the correct indexing assignment for each crystal by comparing intensities, but early attempts at simple algorithms based on this idea were not successful. Recently, Brehm & Diederichs (2014 ▸) described a working algorithm for resolving the ambiguity. The algorithm was first demonstrated on the very first SFX dataset, from photosystem I (Chapman *et al.*, 2011 ▸) as processed using a very early version of *CrystFEL*, and worked well despite the low resolution of the data and primitive processing.

In *CrystFEL*, a simpler algorithm related to this one has been implemented. It also uses a clustering approach, but in only one dimension rather than two as described previously (Brehm & Diederichs, 2014 ▸). It is related to *k*-means clustering (Mac­Queen, 1967 ▸) and is similar to the selective breeding method of Kabsch (2014 ▸). The algorithm is as follows:

(1) A random indexing assignment is made for each crystal.

(2) The correlation coefficient between the intensities for each pair of crystals is calculated.

(3) For each crystal in turn

(*a*) the mean of correlation coefficients between this crystal’s intensities and the intensities of all other crystals with the same indexing assignment, *f*, is calculated,

(*b*) the mean of correlation coefficients between this crystal’s intensities and the intensities of all other crystals with the opposite indexing assignment, *g*, is calculated, and

(*c*) the indexing assignment of the current pattern is swapped if *g* > *f*.

(4) Step 3 is repeated, looping back to the first crystal once the last one has been processed, until the indexing assignments no longer change.

Two sets of reflection intensities from crystals will always exhibit a positive correlation coefficient, provided they cover a large enough resolution range. This is because low-resolution reflections are always the strongest and high-resolution reflections always the weakest, even if the two sets of intensities come from otherwise completely unrelated orientations. To account for this, the correlation coefficients are calculated for a restricted resolution range such as 40 Å up to 4 Å. This resolution range must be set by the user, since the optimum range varies for each dataset.

As with the previous algorithm, *a priori* knowledge of the actual reindexing operation is not required. The algorithm sorts the individual partial datasets from each crystal into two groups with strong intra-group correlation but low inter-group correlation. However, if the reindexing transformation is known (or can be guessed, as is usually the case), the effectiveness can be improved by including in *f* all of the correlation coefficients between the current crystal and the other crystals with the opposite indexing assignment, after reindexing the reflections of the other crystals according to the ambiguity operation.

This algorithm is implemented in the new program *ambigator*, which was added to *CrystFEL* in version 0.5.3. The *ambigator* program starts from the data ‘stream’ as produced by *indexamajig* and outputs a new stream in which the reflections have been reindexed correctly. The calculation of correlation coefficients is the most computationally intensive part of the procedure and is performed by a user-specified number of threads in parallel to accelerate it. If the number of crystals is large, it is not usually necessary to compute all correlation coefficients to resolve the indexing ambiguity: the user can specify a maximum number of correlation coefficients per crystal. Doing so essentially reduces the complexity of the algorithm from 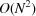 to 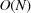, where *N* is the number of crystals. The program usually executes in a small number of minutes for a typical SFX dataset of around 10 000 crystals on a current desktop computer. The program also offers the option of saving the matrix of correlation coefficients in HDF5 format, for use in analysis methods such as hierarchical clustering (Zeldin *et al.*, 2015 ▸).

Some systems, notably those in space groups *P*3, 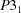 and 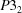, exhibit two indexing ambiguities instead of just one. The two indexing ambiguities lead to a total of four possible indexing assignments. Whereas the previous algorithms handled this situation by clustering the data in three dimensions instead of two, the new algorithm can only be applied to one ambiguity at a time. Nevertheless, the situation can still be handled by running the algorithm twice, first to resolve one of the two ambiguities, then again on the reindexed results from the first run, this time resolving the second ambiguity and hence distinguishing between the four possible indexing assignments.

**Figure 3:**
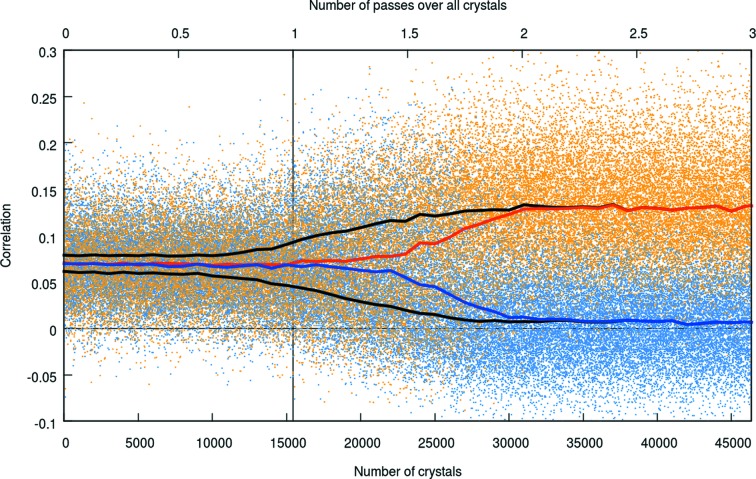
Separation of snapshots into clusters when resolving an indexing ambiguity on the original data stream from Chapman *et al.* (2011 ▸). Orange and blue dots, respectively, show the values *f* and *g* for each crystal. The red and blue lines show the smoothed average values of *f* and *g*, respectively. The upper and lower black lines show the smoothed average values of whichever of *f* or *g* is greater or lower, respectively, for a particular crystal. The algorithm operates by considering each crystal in turn and passing over the entire dataset multiple times. Accordingly, the horizontal axis is labelled twice, once at the bottom of the graph and once at the top, to show both the number of crystals used and the number of passes over the dataset. In this run, the entire dataset (15 445 crystals) was passed over three times, as shown in the top right.

The algorithm as implemented in *ambigator* has been tested on the original data stream from the very first SFX dataset (Chapman *et al.*, 2011 ▸), *i.e.* directly using the results of the original indexing and integration which was performed for this experiment. After a simple text reformatting operation, the data stream from the very early version of *CrystFEL* could be used directly by *CrystFEL* version 0.6.1. Fig. 3 ▸ shows the resulting correlation coefficients. In this graph, orange and blue points, respectively, represent the values of *f* and *g* for the crystal being considered in step 3 of the algorithm. At the start of the process (the left-hand edge of the graph), the orange and blue points overlap with one another. As the algorithm progresses, it visits each crystal in the dataset before starting again from the first crystal, passing over each crystal three times in this case. As would be expected for correct indexing assignments, the values of *f* become systematically higher than those of *g*, both for each individual crystal and in their overall values. The orange and blue points therefore separate into two clusters, which can be seen as the almost complete separation of the orange points from the blue points at the right-hand end of the graph.

The success of this approach probably lies in the fact that it considers the mean of the correlation coefficients between each set of intensities and many other sets, rather than considering only one such correlation coefficient against a reference set of intensities. We have not found it to be necessary to interleave this algorithm with rounds of other data quality enhancements such as scaling or post-refinement as described below — a single run of the algorithm on the integrated intensities, prior to merging with scaling, is sufficient. The restriction of the resolution range over which the correlation coefficients are calculated also appears to be an important contribution to a successful resolution of an indexing ambiguity.

## 5. Prediction refinement   

The final two sections of this article concern modelling the diffraction processes that relate the crystal structure factors to the intensities of the spots observed in the diffraction pattern. Factors that affect this relationship include the intensity of the incident X-ray beam, the size and quality of the crystal, the polarization of the incident and diffracted X-ray beams, the partiality of each reflection (Rossmann *et al.*, 1979 ▸), effects from fast ionization dynamics (Son & Santra, 2011 ▸), the energy spectrum of the incident X-ray beam, and the response of the detector. Correctly modelling these factors and then compensating for them, for example by using a linear scaling factor to account for variations in the incident beam intensity, should reduce the variance of the intensity measurements for a particular reflection and hence result in more precise merged intensities. Equivalently, this should allow a smaller number of crystals to be used to achieve an acceptable level of precision. This has been a very active field in the few years that have passed since the first SX experiments. The enhancements described include refinement of the crystal parameters prior to integration (Sauter *et al.*, 2014 ▸; Ginn, Messer­schmidt *et al.*, 2015 ▸), an accurate refinement of the detector geometry (Ginn, Brewster *et al.*, 2015 ▸; Yefanov *et al.*, 2015 ▸), outlier rejection (Ginn, Brewster *et al.*, 2015 ▸), scaling of the reflection data including both linear and Debye–Waller terms (Sauter, 2015 ▸; Uervirojnangkoorn *et al.*, 2015 ▸), and the inclusion of reflection partiality. All these improvements are implemented within *CrystFEL*.

The first method proposed for SFX data analysis involved the selection of pixels to integrate based on the proximity of the corresponding location in reciprocal space, under the assumption of a monochromatic X-ray beam, to a reciprocal lattice point (Kirian *et al.*, 2010 ▸). This was referred to as the ‘Monte Carlo’ integration method. Since the very first released version of *CrystFEL* (version 0.3.0), integration has been performed using a more conventional method in which a shoe box summation with a fixed radius in pixels is performed around the ‘predicted’ reflection location (White *et al.*, 2013 ▸). More recent versions of *CrystFEL* include two-dimensional profile fitting (Rossmann, 1979 ▸) as an optional alternative method for spot integration. No version of *CrystFEL* has, by default, used the Monte Carlo integration method exactly as it was initially described, although it was available as an option in versions prior to the first generally released version. Nevertheless, we use the term ‘Monte Carlo’ to refer to the process of merging reflection intensities without accounting for the complicated relationship between the crystal structure factors and the intensities observed in the diffraction patterns.

Before version 0.6.1, *CrystFEL*’s indexing and integration program *indexamajig* accepted the indexing solution from the indexing programs [initially *DirAx* (Duisenberg, 1992 ▸) and *MOSFLM* (Powell *et al.*, 2013 ▸), but now also including *XDS* (Kabsch, 1988 ▸) and a new algorithm known as ‘asdf’ built into *CrystFEL* itself] and used it directly to calculate the spot locations for integration. Even if the Monte Carlo merging method is used, in which most of the factors affecting the diffracted intensity are neglected, a model is still required to choose (or ‘predict’) which reflections appear in the diffraction pattern. Effectively, reflections that should be excited according to the model are assigned partialities of 1 and all others are assigned 0. Calculating more accurate partialities could therefore be considered as a more advanced way of making this selection of reflections. One way to do this is by minimizing a residual based on the intensities themselves, which is the classic ‘post-refinement’ method (Rossmann *et al.*, 1979 ▸) and has been described several times in relation to SX data (White, 2014 ▸; Kabsch, 2014 ▸; Sauter, 2015 ▸; Uervirojnangkoorn *et al.*, 2015 ▸; Ginn, Brewster *et al.*, 2015 ▸; Kroon-Batenburg *et al.*, 2015 ▸). Another method to improve the selection of reflections is by minimizing a residual involving the reflection positions relative to observed spots and their distance from the exact Bragg condition (Kabsch, 2014 ▸; Ginn, Messerschmidt *et al.*, 2015 ▸; Sauter *et al.*, 2014 ▸). A combination of the two methods is possible, as was described by Sauter (2015 ▸) and Uervirojnangkoorn *et al.* (2015 ▸), but the two methods are kept separate in *CrystFEL*, where the second method is known as ‘prediction refinement’ because it is concerned with optimizing the prediction of reflections prior to integration.

In *CrystFEL*, prediction refinement is performed by first selecting the observed spots that correspond to the crystal lattice, excluding those which arise from other overlapping diffraction patterns or other sources. This is performed by calculating the closest integral Miller indices for each spot using the basis vectors of the lattice (even if the indices are very far from integral values), then calculating the predicted location for that reflection in the pattern and including only those which fall within ten pixels of the observed spot. This is followed by sorting the reflections into order of increasing values of the mean of the upper and lower ‘excitation error’ values, *r* (White, 2014 ▸). The list is then passed over, and the gradient 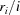 calculated for each reflection *i*. Each reflection *j* with 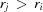 is then examined in turn, in order of increasing 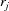, and the list truncated at the first reflection for which
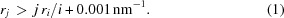
This procedure essentially finds the first abrupt increase of *r*, which appears to herald the end of the correct spot–reflection pairings, and filters out outlying points in a robust manner.

Once the spots have been selected, the following residual is minimized in a nonlinear least-squares procedure which varies the lattice basis vectors and the centre of the diffraction pattern on the detector:
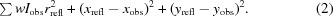
Here, 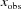 and 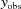 are the positions of an observed spot on the detector in the laboratory coordinate system, 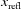 and 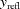 are the positions of the spot as predicted by the diffraction model, 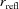 is the mean of the upper and lower ‘excitation error’ values for the reflection, 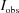 is the intensity of the spot divided by the maximum spot intensity found in the pattern, and 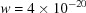. The weighting factor is needed to bring the contributions from excitation error (which is measured in reciprocal metres and has order of magnitude 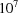) and spot position (which is measured in metres and has order of magnitude 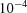) onto approximately the same scale.

**Figure 4:**
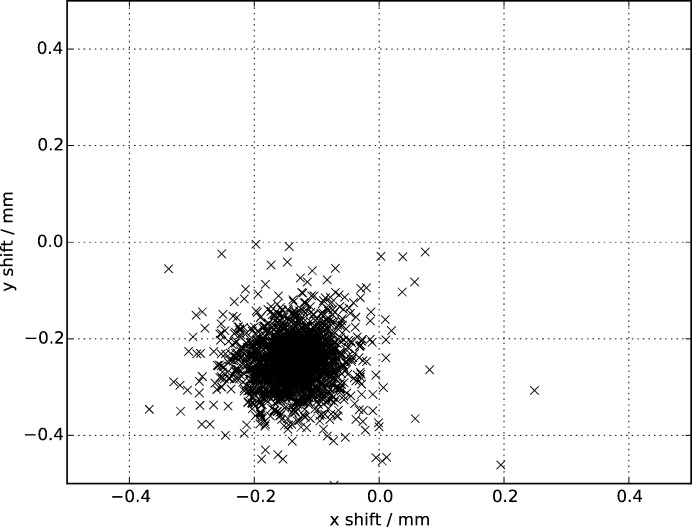
Scatter plot of in-plane shifts to be applied to the entire detector, as determined by the prediction refinement procedure. A clear shift of 0.29 mm, corresponding to 2.6 pixels for this detector, is apparent.

This minimization takes very little additional time and has been found to improve the self-consistency figure of merit 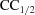 on its own (see §6). The updated beam centre positions generated for each crystal are stored in the data stream and can be used to update the detector geometry in addition to the main geometry refinement tool *geoptimiser* (Yefanov *et al.*, 2015 ▸). Fig. 4 ▸ shows the required offsets for a previously published dataset for the 5-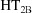 receptor (Liu *et al.*, 2013 ▸), which in this case shows a clear offset corresponding to 2.6 pixels. This plot was calculated using the results of indexing with a target unit cell, but the updated beam centre positions have useful values even on an initial indexing run without reference lattice parameters.

The diffraction model in current versions of *CrystFEL* has been described previously (White *et al.*, 2013 ▸; White, 2014 ▸). It makes use of several parameters for selecting reflections: the beam convergence angle, the bandwidth of the X-ray beam and a notional ‘size’ of the scattering density that surrounds each reciprocal lattice point (‘reciprocal space profile radius’). These parameters can be set manually if accurate values are known; otherwise the convergence angle and bandwidth are set to small values and the profile radius is determined automatically as follows. First, reflections arising from the crystal are selected as described above. Then, the reciprocal space profile radius is set such that 98% of the spots that were assigned indices are predicted. Despite having no component that varies significantly with increasing resolution, we have found that this method gives good matches with the observed spots in most cases. The method could easily be extended to optimize other parameters such as the bandwidth, beam convergence angle or crystal mosaicity.

Another method was recently described for a similar refinement in which the reciprocal space profile radius was minimized directly by adjusting the orientation (Ginn, Messerschmidt *et al.*, 2015 ▸). It can easily be demonstrated that the prediction refinement step has a similar effect by comparing the profile radii before and after the refinement, both of which are stored in the data stream. For the 5-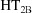 data mentioned above, the radius was reduced for the majority (68%) of crystals, and for nearly half (48%) of them the radius was reduced by more than 10%.

Finally, since version 0.5.3, *CrystFEL* automatically attempts to estimate the resolution to which each crystal diffracts. For this purpose *CrystFEL* considers a spot to be accounted for by a lattice if the Miller indices calculated for the spot using the basis vectors are within 0.25 of integral values. The 98th percentile of the scattering angles of these spots is then taken as a conservative estimate of the resolution limit. *CrystFEL* offers the option of restricting integration to reflections lower than the limit, or extending to a resolution limit higher by a user-specified value (which can even be negative to restrict the resolution further, if required). This cutoff can be performed equally well at the integration or merging stages. Performing the cutoff at the integration stage results in a much smaller output data stream and therefore increases the speed of later processing, whereas performing it at the merging stage allows different scenarios to be explored without repeating the whole integration stage. We have found this method of estimating the resolution to be very robust, and it has increased the quality of the resulting density maps in some cases (Zhang *et al.*, 2015 ▸). The default behaviour is for no resolution cutoff to be applied. Regardless of whether or not the user opts to actually perform the resolution cutoff, the conservative estimated resolution is written into the output data stream, allowing datasets to be quickly and meaningfully compared.

**Figure 5:**
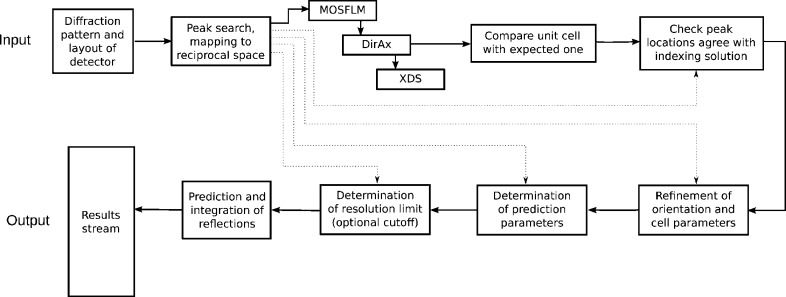
Flow diagram of processing for one frame of data within *indexamajig*. Several indexing methods can be tried in turn, in a user-specified order. For the purposes of illustration, the *MOSFLM*, *DirAx* and *XDS* methods have been selected here, with *DirAx* being the first to produce an indexing solution. The subsequent stages of checking and refining the result can be disabled by the user if required.

The overall flow of the processing of a single frame of data by *indexamajig* is shown in Fig. 5 ▸, which can be compared with the flow diagram for earlier versions (White *et al.*, 2012 ▸). The information from the peak search is used in almost all stages after indexing, with the notable exception of the final prediction and integration stage, which is performed from scratch using the diffraction model constructed and refined in the earlier stages.

## 6. Scaling and post-refinement   

Since the first release version, *CrystFEL* has offered two programs for merging individual reflection intensities into a final dataset which can be exported and used for the subsequent stages of structural analysis. The first is *process_hkl*, which implements a relatively simple merging strategy that takes the average of the individual intensity measurements for each symmetrically unique reflection, after applying corrections for polarization of the incident and diffracted beams. It offers an option for scaling of the intensities that is performed by merging the data twice, the second time multiplying each intensity by the scale factor which gives the best fit to the merged intensities determined on the first pass.

The second merging program is *partialator*, which has much greater capabilities including a more advanced scaling algorithm based on multiple passes over the data. It also offers the possibility of carrying out partiality correction and post-refinement. In versions of *CrystFEL* prior to 0.6.1, none of the algorithms in *partialator* were sufficiently stable to be applied to real experimental data, although they worked for demonstration purposes on simulated data (White, 2014 ▸). As of version 0.6.1, the scaling and merging algorithms in *partialator* have been improved and are now recommended for routine use on real data, having been tested on many datasets. The partiality correction and post-refinement algorithms continue to be under development.

The scaling of reflection intensities in *partialator* is achieved using least-squares minimization on a logarithmic residual, as for the procedure described by Kabsch (2014 ▸). The algorithm starts by merging the intensities from all crystals to make a ‘reference’ dataset. Then, for each pattern, the residual to be minimized is
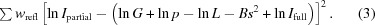
Here 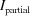 is the intensity of the reflection as measured from the diffraction pattern after polarization correction, *G* and *B* are the linear and Debye–Waller scaling terms, respectively, *L* is the Lorentz factor (currently set to 1), *p* is the estimated partiality of the reflection, 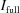 is the merged intensity of reflection, 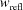 is a weighting factor (also set to 1), and 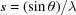, where θ is half the scattering angle and λ is the X-ray wavelength. The sum is over all reflections from one crystal in one snapshot, and minimization is performed by varying *G* and *B*. The advantages of a logarithmic residual are that it improves the numerical stability of the algorithm because very small values of 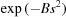 are avoided, weights reflections approximately equally across the whole resolution range despite very large variations in intensity, and guarantees that the resulting value of *G* will be positive. A special consideration when using the logarithmic residual is that reflections with zero or negative values of 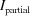 or 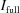 cannot be included.

The least-squares minimization is linear in both 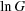 and *B* and can therefore be performed in one iteration per crystal. Crystals with 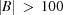
 Å^2^ are rejected, although this cutoff can be changed by the user if required. Once the best values have been determined for all crystals, the intensities from all crystals are merged once more to produce an updated reference dataset, and the process is repeated a user-specified number of times. If the number of parameters to be refined exceeds the number of reflections included in the refinement calculation for any crystal, that crystal is excluded from all further processing.

**Figure 6:**
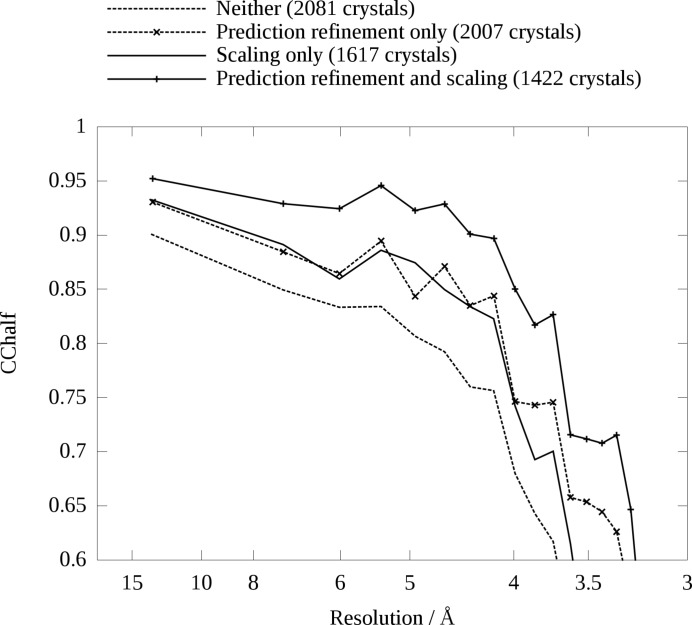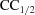 values for the same SFX dataset as is shown in Figs. 1 ▸ and 4 ▸, with and without prediction refinement and scaling.

Fig. 6 ▸ shows the value of the self-consistency figure of merit CC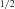 for four cases of data processing applied to a previously published dataset (Liu *et al.*, 2013 ▸): prediction refinement only, scaling only, both and neither. Reflections containing any pixel with a value higher than 14 000 detector units before background subtraction, the approximate saturation value of the detector used for this experiment, were excluded. Reflections were merged up to 0.3 nm^−1^ above the conservatively estimated resolution limit for each crystal. Where scaling was used, three iterations of scaling were performed. Partiality modelling and post-refinement were not performed, *i.e.* partialities were fixed as 1 for all reflections. The number of crystals used in each case varies because of different rejection criteria: 2081 crystals were included for ‘Neither’, 2007 for ‘Prediction refinement only’, 1617 for ‘Scaling only’ and 1422 for ‘Prediction refinement and scaling’. When prediction refinement is used, which is the default in *CrystFEL* since version 0.6.1 but can be disabled if required, crystals are rejected if fewer than ten spots could be assigned indices as described above. When scaling is used, crystals are rejected if *B* is too large, as also described above. Despite the decreasing number of crystals, CC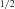 increases as the more sophisticated analysis schemes are applied. The improvement occurs across all resolution shells, and the apparent resolution of the data, as judged by the point where CC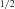 falls off very rapidly, increases by about 0.5 Å. The overall CC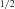 increases from 0.878 for processing without prediction refinement and scaling, through 0.921 for prediction refinement alone, to 0.957 for prediction refinement and scaling combined.

In addition to scaling, *partialator* implements post-refinement as previously described (White, 2014 ▸). So far we have found the improvements from this to be small and counteracted by crystals being rejected as a result of the post-refinement calculation diverging. However, the improvements from the prediction refinement and scaling shown here compare favourably with the results found by other authors for partiality-based analysis schemes applied to SFX data. Sauter (2015 ▸) found an improvement of CC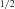 from 0.872 to 0.902 with 12 550 crystals when moving from a scheme similar to *CrystFEL*’s prediction refinement to one including individual *B* factors, partiality correction and post-refinement. Uervirojnangkoorn *et al.* (2015 ▸) found an improvement of CC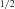 from 0.777 to 0.935 with 2000 crystals of thermolysin, again when moving from a prediction refinement scheme (the one included in *cctbx.xfel*; Hattne *et al.*, 2014 ▸), without scaling, to a full scaling and post-refinement scheme. Their post-refinement scheme includes individual *B* factors as well as aspects similar to the prediction refinement scheme presented here, but they did not report results for these intermediate stages on their own. The same authors found an improvement from 0.918 to 0.982 with 757 crystals of myoglobin when moving from *cctbx.xfel*’s prediction refinement to full scaling and post-refinement, also reporting CC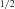 = 0.957 for a more rudimentary scaling scheme without individual *B* factors and without partiality correction and post-refinement. Ginn, Messerschmidt *et al.* (2015 ▸) found an improvement of CC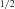 from 0.972 to 0.983 when applying partiality correction to 5787 crystals of a viral polyhedrin, having already refined the orientations of the crystals and scaled (without individual *B* factors). Our result that the additional improvement, over that from the prediction refinement and scaling stages, from correcting partialities is small therefore appears to be compatible with the previous results. Nevertheless, we by no means exclude that our absence of large improvements with partiality-based schemes is due to a deficiency in our implementation, and expect that further improvements in the partiality correction and post-refinement algorithms in *CrystFEL* will lead to it making a more significant improvement. We leave this as the subject for future work, and also acknowledge that CC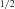 should not be the sole quantifier of SFX data quality.

The *partialator* program performs both the scaling and post-refinement steps including cross-validation similar to that commonly performed during structural refinement (Brünger, 1997 ▸). Five percent of the reflections in the input dataset are excluded from the least-squares minimization procedures, and the residual is calculated separately for these reflections to give a ‘free residual’ which is displayed on screen. The scatter plots of ‘observed’ and calculated partiality (White, 2014 ▸), which can be used to visually assess the effectiveness of partiality correction and post-refinement, are also calculated using the ‘free’ reflections.

Another important feature of *partialator* is the ability to split datasets into sections after scaling and post-refinement. This feature is intended for use when performing a time-resolved serial crystallography experiment [such as those recently described by Tenboer *et al.* (2014 ▸) and Barends *et al.* (2015 ▸)] or an isomorphous replacement experiment (Yamashita *et al.*, 2015 ▸). Here, an extra input file can be provided to *partialator* which gives a ‘dataset identifier’ for each diffraction pattern. Diffraction patterns are identified using either the filename of the original image or the event descriptor (see §3). The dataset identifier can be any sequence of letters or numbers and might represent a time delay (in a time-resolved experiment) or have a value such as ‘native’ or ‘derivative’ (in an isomorphous replacement experiment). All crystals will be scaled and post-refined as described above, but in addition to being merged together to form an overall dataset, they will be merged separately in groups, where each group consists of crystals from images with identical dataset identifiers. Therefore, all of the datasets will be on the same scale, and any potential concerns about the uniqueness of the result of the post-refinement are greatly reduced. For each merged dataset, the crystals will also be randomly split into two half-datasets which will be merged separately. Self-consistency figures of merit for the datasets can be calculated by comparing these split–merged datasets using *compare_hkl*. A pair of split–merged half-datasets is, of course, also written for the entire dataset, regardless of whether or not the custom dataset splitting feature is used. A further advantage of this feature is that, if an indexing ambiguity exists, it can be resolved using a single run of *ambigator* on the entire combined stream and no further checks need to be performed.

## 7. Conclusions   

This article has described some of the most important recent changes in *CrystFEL*, which together constitute a significant enhancement of the software over the first released version 0.3.0 (White *et al.*, 2012 ▸). Improvements have been made in all areas of the suite, including the user interface and data handling and analysis algorithms. All changes, including the developmental changes between versions dating all the way back to the very first lines of code, can be retrieved from the public version control repository, for which details can be found on the *CrystFEL* web site at http://www.desy.de/~twhite/crystfel.

Future work will include further improvements across the suite, in particular to develop the prediction refinement, scaling, post-refinement and diffraction modelling with the aim of fully understanding the factors affecting data quality in an SFX experiment.
